# Cytogenetics and Molecular Investigations detect a Mosaic Variant of Turner Syndrome only Suspected by Non-Invasive Prenatal Testing: Two Case Reports with Negative Ultrasound Examinations

**DOI:** 10.25122/jml-2020-0092

**Published:** 2020

**Authors:** Francesco Libotte, Sonia Lorena Carpineto, Claudio Dello Russo, Antonella Viola, Katia Margiotti, Fabrizia Restaldi, Antonio Novelli, Alvaro Mesoraca, Claudio Giorlandino

**Affiliations:** 1. Department of Genetics, Altamedica Fetal Medical Centre, Rome, Italy; 2. Department of Genetics, Bambino Gesù Children’s Hospital, Rome, Italy; 3. Department of Prenatal Diagnosis, Altamedica Fetal Medical Centre, Rome, Italy

**Keywords:** Counselling, mosaicism, non-invasive prenatal test, prenatal diagnosis, Turner syndrome

## Abstract

Prenatal testing has been moving towards non-invasive methods to determine fetal risk for genetic disorders. Numerous studies have focused the attention on common trisomies; although the detection rate (DR) for trisomy 21 is high (over 95%), the accuracy regarding the DR for trisomies 13 and 18 has come under scrutiny. The testing has been applied to sex chromosome aneuploidies, but many studies have shown that it is not as effective as it is for common trisomies. Although non-invasive prenatal test (NIPT) has become a standard screening procedure for all pregnant women, invasive sampling procedures remain important in confirming NIPT-positive findings. In the present study, we report discordant results of Turner syndrome (TS) mosaicism between NIPT and karyotyping. A 35-year-old pregnant woman underwent NIPT, and a probable risk for Xp deletion was indicated. Subsequently, amniocentesis was performed. The karyotype was identified as mos 45,X [28]/46,X,i(X)(q1.0)[5]. In the second case, a 33-year-old woman underwent amniocentesis after a positive NIPT that indicated a probable risk for monosomy X. The result was mos 45,X [8]/46,XY[8]. Since NIPT is a screening test, the possibility of false-positive or false-negative results should always be considered. We underline the importance of pre/post detailed counseling. Furthermore, women with abnormal NIPT results should undergo immediate amniocentesis that remains the only tool for a correct diagnosis of sex chromosome aneuploidies.

## Introduction

At first, prenatal diagnosis for fetal chromosome abnormalities was made by amniocentesis and subsequently by chorionic villus sampling (CVS). This diagnostic test requires an invasive procedure; however, in the last decade, molecular genetic technologies have developed a non-invasive prenatal testing (NIPT) through the analysis of the cell-free fetal DNA (cfDNA) in maternal plasma [1, 2]. CfDNA isolated from maternal blood derives from the normal cellular turnover of maternal cells. A little fraction is derived from the outer trophoblast cell layer of the placenta in a pregnant woman, which reflects the fetal genotype [3, 4]. The placenta and fetus develop from the same zygote, and the chromosomal complement is the same. In 2% of CVSs, the chromosomal abnormalities are confined to the placenta, a phenomenon known as confined placental mosaicism (CPM) [5, 6]. In 2011, the first tests to detect Down syndrome were launched in China and the United States of America, quickly followed by tests for additional fetal aneuploidies [7]. It has been clearly demonstrated that all NIPT methods are effective for trisomies 21, 18, and 13 [8]. New molecular genomic technologies have allowed using the test to investigate submicroscopic copy number variations (CNV) and various monogenic disorders [9]. Currently, NIPT is used to determine the sex of the fetus and, consequently, sex chromosome aneuploidies (SCA). Many reports underline NIPT’s non-effectiveness for SCA so that the mosaicism for the gain or loss of the X chromosome needs to be further evaluated.

Turner syndrome (TS) is one of the most common chromosomal disorder; it is characterized by numerical or structural abnormalities of the X chromosome and has an estimated incidence of one in 2,500 girls born alive. The clinical features, which occur in more than 90% of TS cases, are variable: short stature and gonadal dysgenesis, typical dysmorphic stigmata, and renal, cardiac, skeletal, endocrine and metabolic abnormalities [10, 11]. About 60% have a 45,X karyotype, and the rest are either mosaics 45X/46XX or have a variety of structural defects of the X chromosome [12, 13]. The gene candidate for short stature is localized in the pseudoautosomal region 1 (PAR1), in the short arm of the X chromosome [14, 15], and it is designated as the short stature homeobox-containing (SHOX) gene. Identified through genotype/phenotype correlations in X/Y abnormalities, Xp22.3 SHOX (X) and Yp11.3 SHOX (Y) [16, 17], the gene is expressed on both the inactivated X chromosome and the activated X or Y chromosome, thereby escaping X inactivation.

## Case report 1

We report the case of a 35-year-old pregnant woman without ultrasound evidence and without a remarkable family history who underwent counting-based non-invasive prenatal testing (NIPT). The blood was sampled for NIPT at 12 weeks of gestation. The fetal DNA fraction in the maternal plasma sample was 13.7%, with a chromosome Z-score of -5.452, indicating a probable risk for Xp deletion.

Subsequently, after a sonographic examination at 15 weeks and 5 days of gestation, which was negative, amniocentesis was immediately performed.

**Figure 1:**
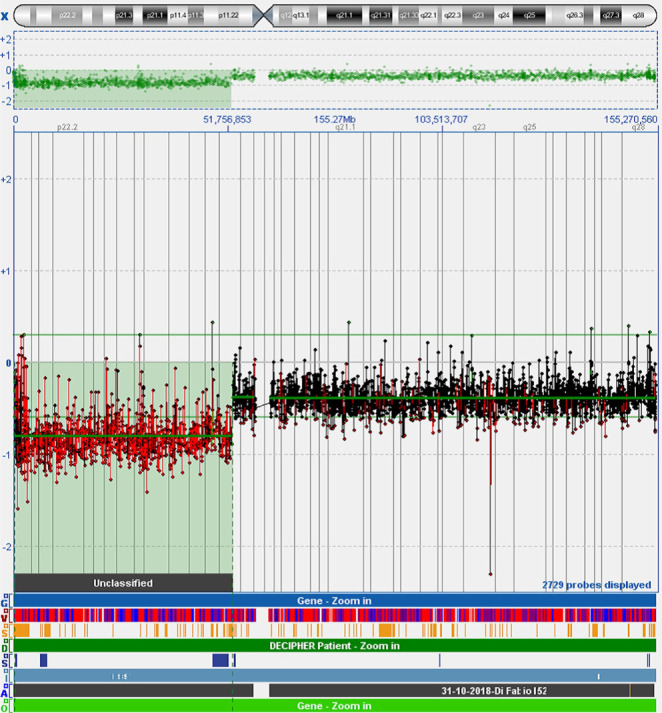
Chromosome microarray analysis on amniotic cells. The results demonstrated a single copy gain of 52.27 Mb on the Xp arm.

Microarray-based comparative genomic hybridization (array-CGH) analysis was performed on the DNA extracted from amniotic cells. Array-CGH was executed by using a 180K platform (Agilent Technologies, Santa Clara, CA) according to the manufactures’ protocol. The array-CGH analysis showed the whole chromosome X deletion, including Xp deletion and Xp11.22-Xqter. The difference is that deletion in the latter region is mosaic; a 52.27 Mb deletion of Xp22.33p11.22(312708_52587526)x1 [18, 19] confirmed a single-copy loss of the Xp arm in accordance with the NIPT results (Figure 1).

**Figure 2:**
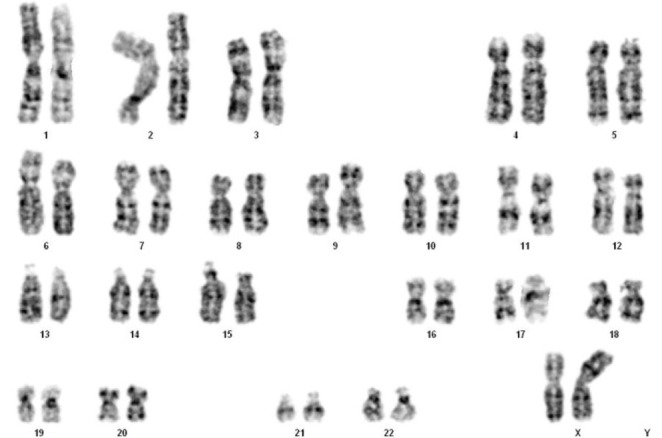
GTG-banding showed a karyotype of 46,X,i(X)(q10).

Prenatal cytogenetic analysis was performed from the amniotic fluid using culture cells according to standard G-banding techniques. Fetal karyotype was 45,X in 28 examined metaphases and 46,X,i(X)(q10) in 5 of 33 cells (Figure 2). Analysis of amniotic cells confirmed the presence of Xp deletion and presented another structural aberration, isochromosome Xq. The karyotype was identified as mos 45,X[28]/46,X,i(X)(q1.0)[5], in accordance with the array-CGH result.

Subsequent chromosome analysis of lymphocyte cultures of both parents was performed, and both were normal.

Fluorescent in situ hybridization (FISH) experiments were performed during metaphase spreads for further confirmation of the diagnosis. Analysis of metaphases with the SHOX probe [SHOX(Xp22.33)(SR)/DYZ1(Yq12)(SG)/DXZ1(Xp11.1-q11.1)SA] was tested in accordance with the manufacturers’ instructions. FISH analysis of the 100 nuclei showed the presence of 2 different cell lines. About 85% of the cells exhibited monosomy of chromosome X (45X), 15% of the cells presented two centromeres of the X chromosome, and only one signal of the SHOX gene. The placenta study was impossible to execute because the sample was not unavailable.

## Case report 2

A 33-year-old primiparous woman without medical indications came to our clinic for a non-invasive prenatal diagnosis at 11 weeks of gestation. Fetal DNA fraction in the maternal plasma sample was 9.97% with a chromosome Z-score of -7.69, indicating a probable risk for monosomy X.

Subsequently, she presented to our clinic at 17 weeks of gestation, and a sonographic examination was performed. The fetus was found to be appropriate for gestational age. The structural analysis failed to show any of the abnormalities associated with Turner syndrome but was noticed that the sex fetus was male. In light of these findings, the patient was offered a diagnostic amniocentesis. Prenatal cytogenetic analysis was performed using culture cells. Fetal karyotype was 45,X in 8 examined metaphases and 46,XY in other 8 examined metaphases. The karyotype of the fetus was designated as mos 45,X[8]/46,XY[8]. The karyotype of both parents was negative.

**Figure 3:**
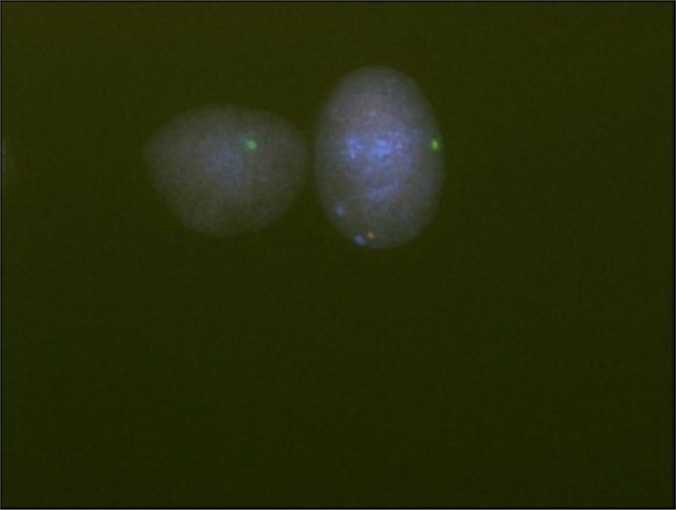
FISH for interphase nuclei from the amniotic fluid. A positive SRY signal indicated by the red light is found at the terminal Y p-arm. The centromere of the X chromosome is shown by the green light. Interphase nuclei: the one with one green signal and the other with red and green signals.

To confirm the presence of the Y chromosome, a FISH analysis was performed using SRY probes, including the SRY gene (Yp11.31 –Sex Determining Region) and the X chromosome centromere (DXZ1). The result was nuc ish (DXZ1x1,SRYx0)[48]/(DXZ1x1,SRYx1)[52]. We found 48/100 nuclei with a centromeric green signal on the X chromosome and 52/100 with an additional red signal on the p-arm of Y chromosome. (Figure 3).

## Discussion

In the first report, we described the case of a 35-year-old secondigravida. There was nothing worthy of special mention regarding her family history and past medical history. Based on the maternal age and negative clinical notices, she underwent NIPT at 12 weeks of gestation after genetic counseling. The NIPT resulted in a positive result for Xp deletion. She immediately presented to our clinic at 15 weeks and 5 days of pregnancy, and a detailed ultrasound examination was performed. The fetus was found to be appropriate for gestational age. Structural analysis of the fetus failed to show any abnormalities associated with TS, such as lymphedema or cystic hygroma. In light of these findings, the patient decided to undergo a diagnostic amniocentesis. Using the array-CGH technique, we verified the deletion of the Xp region. The subsequent prenatal cytogenetic analysis presented a different result: the presence of two cell lines that was confirmed by FISH. The final karyotype was identified as mos 45,X[28]/46,X,i(X)(q1.0)[5].

In the second case, a 33-year-old woman without medical indications underwent NIPT. No aneuploidies were detected in chromosomes 21, 18, and 13, but we found the presence of only one chromosome X. During the follow-up ultrasound examination of the fetus, the external genitalia appeared male; the test was repeated to exclude the false-positive NIPT screening, but no presence of the Y chromosome was reported. The pregnant woman allowed us to investigate these gender discordant results between the NIPT and ultrasound screening. Analysis of amniotic fluid was performed, and the karyotype was identified as mos 45,X [8]/46,XY[8], as subsequently confirmed by FISH analysis.

After counseling, in which TS was associated with the karyotype, both couples decided to terminate the pregnancy.

The studies described in this manuscript compared the efficiency of NIPT with karyotype analysis. In conclusion, every test and diagnostic procedure has its benefits and risk. All the prenatal genetic screenings are designed to decrease the risk related to invasive amniocentesis. NIPT offers better sensitivity and specificity for trisomies 13, 18, 21, microdeletions, and some monogenic disorders [20]. The karyotype analysis of amniotic fluid cells is the main method for detecting fetal sex-chromosome abnormalities and is currently considered the gold standard for the cytogenetic diagnosis. However, these prenatal screening tests are not yet ready to replace completely invasive diagnostic procedures. In fact, NIPT cannot confirm mosaicism. However, on the other hand, if NIPT shows a high risk, it is important to undergo further tests because a pathology might be present [21]. Therefore, we suggest that pregnant women who are interested in NIPT should receive detailed counseling that will explain the benefits and limitations of the test with adequate informed consent. The aim of counseling is to provide sufficient understanding of the test characteristics; it is called an ‘informed choice’ regarding whether a pregnant woman wants to undergo this test, another one, or no test at all [22]. Moreover, we recommend that all women who had positive ultrasound examinations undergo an invasive prenatal test immediately.

## Conclusion

Recent studies show that procedure-associated risks are very low when amniocentesis is performed by experienced clinicians. Invasive testing remains an important part of prenatal care, and the nature of NIPT as a screening test will not change. NIPT is testing the cell-free placenta DNA rather than fetal DNA, so it is not a diagnostic procedure.

## Conflict of Interest

The authors declare that there is no conflict of interest.
